# Vitamin D deficiency is associated with risk of developing peripheral arterial disease in type 2 diabetic patients

**DOI:** 10.1186/s12872-019-1125-0

**Published:** 2019-06-17

**Authors:** Jing Yuan, Pu Jia, Lin Hua, Zhong Xin, Jin-Kui Yang

**Affiliations:** 1 Department of Endocrinology, Beijing Tongren Hospital, Capital Medical University, 1 Dong Jiao Min Xiang, Beijing, 100730 China; 2 Department of Orthopaedics, Beijing Friendship Hospital, Capital Medical University, Beijing, 100050 China; 3 Department of Mathematics, School of Biomedical Engineering, Capital Medical University, Beijing, 100069 China

**Keywords:** Type 2 diabetes, 25-hydroxyvitamin D, Vitamin D deficiency, Peripheral arterial disease

## Abstract

**Background:**

The relationship between vitamin D levels and peripheral arterial disease (PAD) remains unclear. We assessed the association of serum 25-hydroxyvitamin D (25(OH)D) levels with the prevalence of PAD in patients with type 2 diabetes mellitus(T2DM).

**Methods:**

A total of 1018 T2DM patients participated in this cross-sectional study. Serum 25(OH)D levels were measured and risk factors of PAD were recorded. PAD was diagnosed as an ankle-brachial index (ABI) < 0.9.

**Results:**

The mean age of the diabetic patients was 58.59 ± 11.34 years. Of all the patients, only 20.1% had a 25(OH)D level ≥ 20 ng/mL. Compared to patients without PAD, serum 25(OH)D levels were significantly lower in those with PAD (14.81 ± 8.43 vs. 11.55 ± 5.65 ng/mL, *P* < 0.001). The overall prevalence of PAD was 7.7%. From the highest level (≥ 20 ng/mL) to the lowest level (< 10 ng/mL) of serum 25(OH)D, the prevalence of PAD was 2.8, 7.5 and 10.7% respectively. After adjustment for age, sex, body mass index (BMI), smoking status and season, compared to patients with serum 25(OH)D levels ≥20 ng/mL, the odds ratios of PAD in patients with a level of 10 to < 20 ng/mL and < 10 ng/mL was 3.587(95% CI: 1.314–9.790) and 5.540(95% CI: 2.004–15.320), respectively. When further considering the influence of coronary heart disease (CHD), hypertension and cerebral infarction, the ratios changed to 3.824(95% CI: 1.378–10.615) and 5.729(95% CI: 2.028–16.187), respectively. And after further adjustment for the duration of diabetes, glycated hemoglobin (HbA1c) and glomerular filtration rate (GFR), the ratios changed to 3.489(95% CI: 1.100–11.062) and 3.872(95% CI: 1.168–12.841), respectively.

**Conclusions:**

Reduced serum vitamin D levels were associated with an increased risk of PAD in T2DM patients. Randomized interventive clinical studies are required to verify the effects of vitamin D supplementation on PAD.

## Background

Peripheral arterial disease (PAD) is one of the most common complications of diabetes, which leads to a high risk of morbidity and mortality from cardiovascular disease [1]. It affects approximately 8.5 million adults aged ≥40 years in the US [2]. A survey of 21,152 eligible participants in 7 different cities in China indicated that the prevalence of PAD in the male and female natural population was 2.52 and 3.66% respectively [3]. As prevalence of diabetes in China is increasing, so are the risks of its comorbidities. Although most PAD patients are asymptomatic, they are still at an increased risk of developing cardiovascular disease [1].

There are some well-established risk factors for PAD, such as diabetes, older age, smoking status, dyslipidemia and hypertension [4]. Vitamin D is usually considered to have a regulatory role in calcium homeostasis and bone metabolism. Recently, more and more evidence has shown the unconventional roles it plays in the body, including insulin resistance, ß cell dysfunction [5], prevalence of type 2 diabetes mellitus (T2DM) and metabolic syndrome [6, 7] and dyslipidemia [8]. However, at present, the relationship between vitamin D status and PAD remains to be unequivocally elucidated. A number of human studies have suggested that there is a strong relationship between vitamin D deficiency and the prevalence and severity of PAD [9, 10]. Other studies have reported that a low level of serum 25(OH)D might lead to arterial calcification, possibly because it can cause osteoblast-like functions in arterial smooth muscle cells, which result in calcium deposition in arterial walls [11, 12]. Meanwhile, it has also been demonstrated that serum 25(OH)D levels are not associated with arterial stiffness or PAD [13, 14]. Furthermore, there is limited literature regarding what, if any, association exists between vitamin D status and PAD in Chinese T2DM patients.

Therefore, we carried out a cross-sectional study to investigate the relationship between serum 25(OH)D levels and the prevalence of PAD with ankle brachial index (ABI), after adjusting for known risk factors in hospital-based T2DM patients. The results of this study may provide new insights for the likely role of vitamin D in atherosclerotic disease.

## Methods

### Study population

**Fig. 1:**
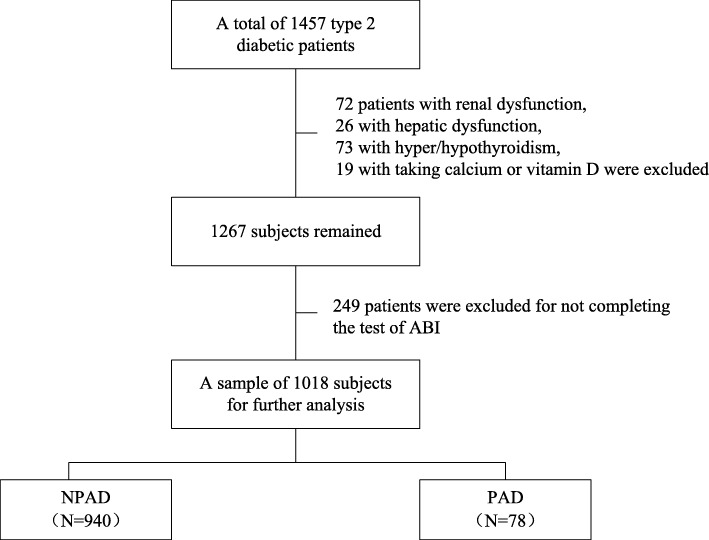
Flowchart of the study. NPAD: no peripheral arterial disease; PAD: peripheral arterial disease

A total of 1457 T2DM patients admitted to the Department of Endocrinology, Beijing Tongren Hospital, Capital Medical University between January 2015 and May 2018 participated in this cross-sectional study. All patients had free exposure to sunlight. Exclusion criteria were: 1) treated with medication that may interfere with bone metabolism, such as bisphosphonates, calcium and vitamin D supplements, calcitonin, estrogen and selective estrogen receptor modulators or corticosteroids; 2) diagnosed with hyperthyroidism, hypothyroidism, hyperparathyroidism, hypoparathyroidism or hypercortisolism; 3) had chronic gastrointestinal diseases, such as gastroduodenal ulcer, inflammatory bowel disease or chronic diarrhea;4) hepatic dysfunction, defined as alanine aminotransferase (ALT) levels ≥2 times the upper reference limit; 5) renal dysfunction, defined as serum creatinine higher than the upper reference limit. Among the 1457 diabetes patients, 72 patients had renal dysfunction, 26 had hepatic dysfunction, 73 had hyper/hypothyroidism, 19 were taking calcium or vitamin D and 249 did not complete the test of ABI, leaving 1018 patients for further analysis. The flowchart of the study was shown in Fig. 1.

### Baseline characteristics of patients

All the patients completed a detailed questionnaire, including the patient’s age, sex, duration of diabetes, season of hospitalization, smoking status, history of hypertension, hyperlipidemia, coronary heart disease (CHD) and cerebral infarction. Smoking status was classified as never, former or current. Patients with blood pressure ≥ 140/90 mmHg or being treated with anti-hypertensive medication were diagnosed as hypertension. Hyperlipidemia was defined as low density lipoprotein (LDL) ≥ 3.5 mmol/L or having history of using lipid-lowering medication. CHD was defined as having a history of coronary stent implantation or coronary artery bypass grafting, showing myocardial ischemia in the EKG, having typical symptoms of angina or myocardial infarction. Cerebral infarction was defined as having a definite disease history, or showing the signs of cerebral infarction in CT or MRI scans. Body mass index (BMI) of each patient was calculated as the weight (kg) divided by the square of height (m^2^).

All subjects underwent retinal photography to determine diabetic retinopathy using a TopconTRC-NW7SF fundus camera (Topcon, Tokyo, Japan) ophthalmic digital imaging system. The results of fundus photography were evaluated and graded as follows: non-diabetic retinopathy (NDR); non-proliferative diabetic retinopathy (NPDR) and proliferative diabetic retinopathy (PDR). A renogram was used to estimate the glomerular filtration rate (GFR). Glycated hemoglobin (HbA1c) was measured by high-performance liquid chromatography (VARIANT, Bio-Rad Lab., Hercules, CA, US). Serum creatinine, uric acid, total cholesterol (TC), triglycerides (TG), high density lipoprotein (HDL) cholesterol, LDL cholesterol concentrations, calcium, phosphate, alkaline phosphatase (ALP) were measured by an automated biochemical analyzer (Beckman company, US). Serum 25(OH)D was detected with an electrochemiluminescence method .

### ABI measurements

Doppler ultrasound (Huntleigh) was adapted. All patients were asked to have a 10 min rest, and maintain a supine position. Systolic blood pressure was measured in the brachial, posterior tibial and dorsalis pedis arteries, with blood pressure cuffs positioned on both sides of the upper arms and legs. ABI was calculated by dividing the higher systolic ankle (posterior tibial or dorsalis pedis) pressure by the higher brachial artery systolic pressure. Patients with an ABI<0.9 were considered to have PAD.

### Statistical analysis

Continuous variables were expressed as mean ± standard deviation ($\overline{\upchi}$ ±s), and the comparison between 2 groups was made using a *t*-test. Categorical measures were expressed as percentage and a chi-squared test was used for analysis. Binary logistic regression analysis was used to analyze the influencing factors of PAD in hospitalized T2DM patients according to 3 Models (Model 1 was adjusted for age, sex, BMI, smoking status and season. Model 2 was adjusted for age, sex, BMI, smoking status, season, CHD, hypertension and cerebral infarction. Model 3 was adjusted for age, sex, BMI, smoking status, season, CHD, hypertension, cerebral infarction, HbA1c, duration of diabetes and GFR). Receiver operating characteristic (ROC) curves were plotted to evaluate the accuracy of the 3 Models for diagnosing PAD. The area under the ROC curve (AUC) and the corresponding 95% confidence intervals were determined at the same time. A simple-to-use nomogram was made to visualize the regression model. SPSS ver. 24.0 software (SPSS Inc., Chicago, IL, US), MedCalc ver. 18.0 software (MedCalc Software, Ostend, Belgium) and R ver. 2.8.1(R foundation for Statistical Computing) with the rms package were used for statistical analyses. A *P-*value <0.05 was considered to be a statistically significant difference.

## Results

### Characteristics of participants

Among the 1018 T2DM patients, the mean age was 58.59 ± 11.34 years and the mean HbA1c value was 8.79 ± 1.81%. There were 564 males (55.4%), with a mean age of 56.47 ± 11.35 years; the mean HbA1c was 8.74 ± 1.81%. There were 454 females (44.6%), with a mean age of 61.23 ± 10.78 years; the mean HbA1c was 8.86 ± 1.82%. Of all the patients, 20.1% had a 25(OH)D level of ≥20 ng/mL, 48.1% had a level of 10 to < 20 ng/mL, and 31.8% had a level of <10 ng/mL. Patients with PAD tended to be older, had diabetes for a longer duration, higher serum creatinine levels and a lower GFR. These patients were more likely to have diabetic retinopathy and a history of hypertension, CHD and cerebral infarction. There were no significant differences in sex, BMI, smoking status, HbA1c levels, serum uric acid or serum lipid metabolism between participants with and without PAD (*P* > 0.05). Additionally, compared to patients without PAD, patients with PAD showed no significant differences in serum calcium, phosphate and ALP (*P* > 0.05) (Table 1).

**Table 1:** Clinic characteristics of type 2 diabetic patients with or without PAD

	NPAD (*N* = 940)	PAD (*N* = 78)	P
Age, years	57.82 ± 11.12	67.88 ± 9.77	0.000
Male, n(%)	526 (56.0%)	38 (48.7%)	0.216
BMI, kg/m^2^	25.58 ± 3.36	26.08 ± 3.62	0.204
Smoking status, n(%)			
never	589 (62.7%)	45 (55.7%)	
former	105 (11.2%)	15 (19.2%)	0.104
current	246 (26.2%)	18 (23.1%)	
Duration of diabetes			
≥10 years	565 (60.1%)	57 (73.1%)	0.024
<10 years	375 (39.9%)	21 (26.9%)	
Hypertension, n(%)	525 (55.9%)	62 (79.5%)	0.000
Hyperlipidemia, n (%)	505 (53.7%)	46 (59.0%)	0.371
CHD, n (%)	201 (21.4%)	37 (47.4%)	0.000
Cerebral infarction, n (%)	119 (12.7%)	20 (25.6%)	0.001
Diabetic retinopathy, n (%)			
NDR	665 (70.7%)	39 (50.0%)	
NPDR	171 (18.2%)	24 (30.8%)	0.001
PDR	104 (11.1%)	15 (19.2%)	
GFR, ml/min	92.22 ± 19.92	75.33 ± 19.90	0.000
HbA1c, %	8.77 ± 1.82	9.12 ± 1.71	0.108
Serum creatinine, umol/L	65.34 ± 15.17	72.77 ± 17.97	0.001
Serum uric acid, mmol/L	329.92 ± 79.93	340.85 ± 82.75	0.253
TC, mmol/L	4.47 ± 0.99	4.55 ± 1.17	0.488
LDL, mmol/L	2.68 ± 0.84	2.74 ± 0.97	0.598
HDL, mmol/L	1.20 ± 0.31	1.02 ± 0.27	0.712
TG, mmol/L	1.85 ± 1.57	1.96 ± 1.23	0.537
Serum 25(OH)D, ng/ml	14.81 ± 8.43	11.55 ± 5.65	0.000
Serum calcium, mmol/L	2.25 ± 0.09	2.26 ± 0.10	0.412
Serum phosphate, mmol/L	1.26 ± 0.17	1.24 ± 0.16	0.314
ALP, mmol/L	73.42 ± 22.2	73.35 ± 23.33	0.978

*NPAD* no peripheral arterial disease, *PAD* peripheral arterial disease, *BMI* body mass index, *CHD* coronary heart disease, *NDR* non-diabetic retinopathy, *NPDR* non-proliferative diabetic retinopathy, *PDR* proliferative diabetic retinopathy, *GFR* glomerular filtration rate, *HbA*1c glycated hemoglobin, *TC* total cholesterol, *LDL* low density lipoprotein, *HDL* high density lipoprotein, *TG* triglycerides, *25(OH)D* 25-hydroxyvitamin D, *ALP* alkaline phosphatase

### The prevalence of PAD

A total of 78(7.7%) T2DM patients had PAD and the cohort was comprised of 38 males (48.7%) and 40 females (51.3%). The prevalence of PAD was gradually increased from patients with the highest (≥ 20 ng/mL), medium (10 to <20 ng/mL) to the lowest (< 10 ng/mL) levels of serum 25(OH)D, which was 2.8, 7.5 and 10.7% respectively (*P* > 0.05). Compared to patients without PAD, serum 25(OH)D levels were significantly lower in subjects with PAD (14.81 ± 8.43 vs. 11.55 ± 5.65 ng/mL, *P* < 0.001).

### Association between 25(OH)D levels and PAD

The association between 25(OH) D levels and PAD was analyzed in 3 Models. After adjustment for Model 1, compared to patients with serum 25(OH)D ≥ 20 ng/mL, the odds ratio of PAD in patients with a level of 10 to <20 ng/mL and < 10 ng/mL was 3.587(95% CI: 1.314–9.790) and 5.540(95% CI: 2.004–15.320), respectively. When further considering the influence of CHD, hypertension and cerebral infarction, the ratio changed to 3.824(95% CI: 1.378–10.615) and 5.729(95% CI: 2.028–16.187), respectively. And after further adjustment for the duration of diabetes, HbA1c and GFR, the ratio changed to 3.489(95% CI: 1.100–11.062) and 3.872(95% CI: 1.168–12.841), respectively (Table 2).

**Table 2:** Odds ratios of PAD by categorical 25(OH)D levels

	Variable	OR	95%CI	P
Model 1	25(OH)D ≥ 20 ng/ml	1(ref)		
	25(OH)D 10 to<20 ng/ml	3.587	1.314–9.790	0.013
	25(OH)D<10 ng/ml	5.540	2.004–15.320	0.001
Model 2	25(OH)D ≥ 20 ng/ml	1(ref)		
	25(OH)D 10 to<20 ng/ml	3.824	1.378–10.615	0.010
	25(OH)D<10 ng/ml	5.729	2.028–16.187	0.001
Model 3	25(OH)D ≥ 20 ng/ml	1(ref)		
	25(OH)D 10 to<20 ng/ml	3.489	1.100–11.062	0.034
	25(OH)D<10 ng/ml	3.872	1.168–12.841	0.027

Model 1 was adjusted for age, sex, BMI, smoking status and season

Model 2 was adjusted for age, sex, BMI, smoking status, season, CHD, hypertension and cerebral infarction

Model 3 was adjusted for age, sex, BMI, smoking status, season, CHD, hypertension, cerebral infarction, HbA1c, duration of diabetes and GFR

*PAD* peripheral arterial disease, *25(OH)D*: 25-hydroxyvitamin D

### Comparison of 3 models for diagnosing PAD

**Fig. 2:**
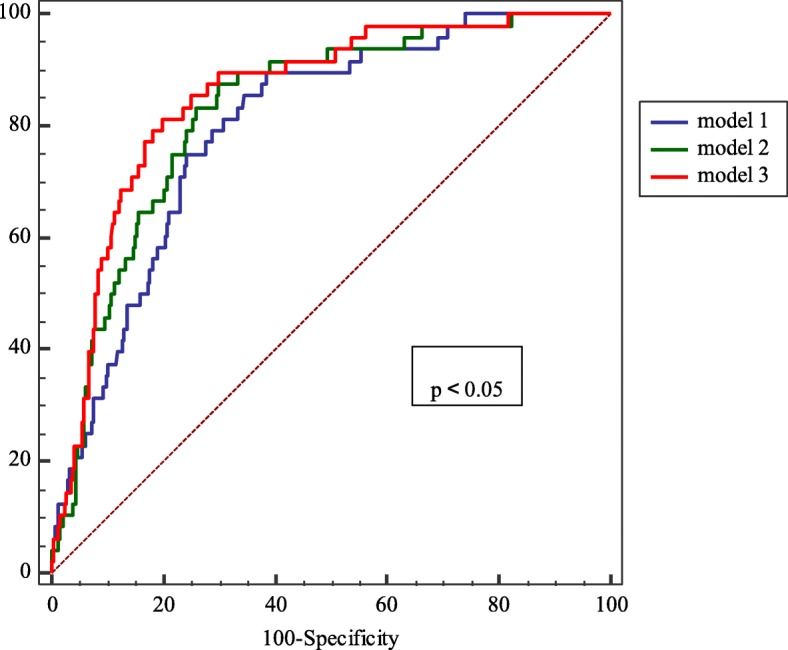
Comparison of the three logistic regression models for diagnosing PAD in patients with T2DM

**Fig. 3:**
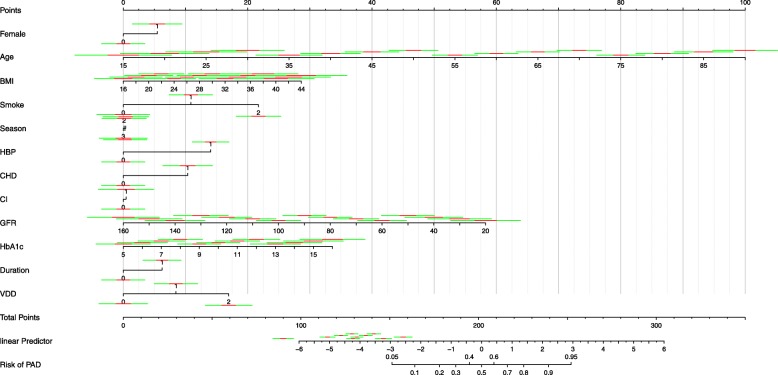
A simple-to-use nomogram for diagnosing PAD. Instructions for using the nomogram: Draw a perpendicular line from the axis of each risk factor to determine the corresponding “POINTS” . The total points of all risk factors are then obtained. After that, draw a line descending from the axis “TOTAL POINTS” until it reaches the axis of “Rik of PAD”. For binary variables (except “Duration”), 0 = no and 1 = yes. For duration categories, 0 = duration of diabetes≥10 years and 1 = duration of diabetes<10 years. For season categories, 0 = spring, 1 = summer, 2 = autumn, 3 = winter. For smoke categories, 0 = never, 1 = former and 2 = current. For VDD categories, 0 = serum 25(OH)D ≥ 20 ng/ml, 1 = serum 25(OH)D 10 to<20 ng/ml and 2 = serum 25(OH)D<10 ng/ml

The AUC areas of Model 1, Model 2 and Model 3 were 0.799 (95%CI: 0.768–0.828), 0.829 (95%CI: 0.799–0.856) and 0.854 (95%CI: 0.826–0.879), respectively (Fig. 2). All the models had a positive value in diagnosing PAD(*P* < 0.0001). There were statistical differences between the 3 Models, and Model 3 turned out to be the most powerful model. A nomogram was developed to visualize the Model 3 that could assess the risk of PAD (Fig. 3).

## Discussion

In this cross-sectional study of 1018 hospitalized T2DM patients, we demonstrated that 79.9% of patients in the study had serum 25(OH)D levels lower than 20 ng/mL, while only 20.1% had levels higher than 20 ng/mL, which means vitamin D deficiency in T2DM patients was very common. The serum 25(OH)D levels in patients without PAD were much higher than those in patients with PAD. In the different vitamin D groups from the highest to the lowest levels, the prevalence of PAD was increased gradually (2.8, 7.5 and 10.7% respectively). Surprisingly, the association of 25(OH)D and PAD was still significant after adjustment for known PAD risk factors and related indexes of diabetes.

Many studies have confirmed an association between vitamin D deficiency and increased prevalence of cardiovascular events or mortality [15, 16]. However, there is a controversy in the relationship between vitamin D deficiency and PAD. A number of studies have demonstrated that low serum 25(OH)D levels are closely linked with PAD. Results from NHANES 2001–2004 [10] analyzed the data of 4839 participants and revealed that after multivariable adjustment, compared to the highest 25(OH)D quartile (≥29.2 ng/mL), the prevalence ratio of PAD for the lowest 25(OH)D quartile (< 17.8 ng/mL) was 1.80 (95% CI: 1.19–2.74). Another study [17] reviewed the medical data of 1435 veterans and concluded that vitamin D deficiency was associated with an increased amputation risk in veterans with PAD. However, other studies did not agree with this conclusion. Veronese N et al [18] selected 3099 participants and found no evidence of any significant association between the incident of PAD and serum 25(OH)D levels after a mean of 4.4 years follow-up, no matter whether adjusting for known PAD risk factors or not. Another cohort study [19] followed 11,789 subjects for over 17.1 years. In this study, it was shown that compared to participants with sufficient 25(OH)D, the hazard ratio of PAD in those with deficient 25(OH)D levels was 1.25 (95% CI: 1.06–1.48) after adjustment for demographic characteristics, BMI, physical activity, and smoking status. However, after further inclusion of cardiovascular risk factors, there was no evident association (OR: 1.15; 95%CI: 0.97–1.37)). Racial differences may be a contributing factor to the contradictory results [9]. To the best of our knowledge, there have been only a small number of Chinese studies in this field [20, 21]. Therefore, more data of Chinese population are urgently needed in future investigations. Our findings revealed that there was a strong association between low serum 25(OH)D levels and the prevalence of PAD after adjustment for various risk factors, strongly suggesting that vitamin D deficiency is an independent risk factor for PAD.

Several possible mechanisms have been described to explain the association between low serum vitamin D levels and the increased risk of developing PAD. In vitro studies, vitamin D levels have shown an association between obesity, diabetes and dyslipidemia [22–24], which are all significant risk factors for PAD. In the present study, even excluding the influence of PAD risk factors, the relationship between serum 25(OH)D levels and PAD was still present, which means maybe some other mechanisms are also involved. First, in vivo studies, active vitamin D calcitriol inhibits endothelial cell activation and TNF-α adhesion molecule expression, which play a role in the various stages of atherosclerosis [25]. Second, vitamin D can modulate and regulate the activity of inflammatory cytokines such as TNF-α and IL-10, and thus influencing the atherosclerotic process [26]. In addition, vitamin D may increase platelet aggregation and thrombogenesis [27]. Importantly, low levels of vitamin D may upregulate the renin-angiotensin-aldosterone system (RAAS) [28],which could stimulate collagen formation, matrix remodeling and vascular hypertrophy, increase oxidant stress, depress nitric-oxide-dependent signaling, and reduce elastin synthesis [29], leading to the development of atherosclerosis.

Nomograms, as statistical tools, have been widely used to aid clinical decisions. Nomograms could help physicians not only to estimate the likelihood of a specific event for an individual subject (i.e. cancer survival time), but also to predict the outcome using combined clinical risk factors [30]. In the present study, we developed a nomogram-illustrated model to assess the risk of PAD in T2DM patients, which may be used in clinical practice and to inform patients about their risk of developing PAD. Furthermore, the nomogram may be applied for preventing PAD, such as losing weight, better control of blood glucose and supplementation of vitamin D. However, more evidence from randomized controlled trials will be required to evaluate the effect of the preventive therapeutic strategies with the risk factors in the nomogram.

There are some limitations to our study conclusions. First, this was a cross-sectional study and residual confounding factors may have remained, so it is difficult to conclude definitive cause-effect relationships. Second, as a single center hospitalized study, the enrolled patients may be different from the actual demographic profile of Beijing. Third, the number of patients in the study was relatively small. And a larger sample size prospective study will be preferable. Fourth, we did not differentiate symptomatic and non-symptomatic patients with PAD in the current study. Finally, ABI was the only measurement used to diagnose PAD. Ultrasonography and angiography of the lower extremities should be conducted in the future studies.

## Conclusions

Reduced serum vitamin D levels increased the risk of PAD in T2DM patients. This association was still strong after adjustment for known PAD risk factors and related indications of diabetes. Considering PAD is a very common and severe complication of T2DM, randomized intervention clinical studies should be carried out to verify the effect of vitamin D supplementation on PAD.

## Data Availability

The data that support the findings of this study are available from the corresponding author upon reasonable request.

## Abbreviations

1,25(OH)2D: 1,25 dihydroxyvitamin D
25(OH)D: 25-hydroxyvitamin D
ABI: Ankle-brachial index
ALP: Alkaline phosphatase
ALT: Alanine aminotransferase
AUC: The area under the ROC curve
BMI: Body mass index
CHD: Coronary heart disease
GFR: Glomerular filtration rate
HbA1c: Glycated hemoglobin
HDL: High density lipoprotein
LDL: Low density lipoprotein
NDR: Non-diabetic retinopathy
NPDR: Non-proliferative diabetic retinopathy
PAD: Peripheral arterial disease
PDR: Proliferative diabetic retinopathy
ROC: Receiver operating characteristic
T2DM: Type 2 diabetes mellitus
TC: Total cholesterol
TG: Triglycerides

## References

[1] Criqui MH, Langer RD, Fronek A, Feigelson HS, Klauber MR, McCann TJ, Browner D (1992). Mortality over a period of 10 years in patients with peripheral arterial disease. N Engl J Med.

[2] Allison MA, Ho E, Denenberg JO, Langer RD, Newman AB, Fabsitz RR, Criqui MH (2007). Ethnic-specific prevalence of peripheral arterial disease in the United States. Am J Prev Med.

[3] Wang Y, Xu Y, Li J, Wei Y, Zhao D, Hou L, Hasimu B, Yang J, Yuan H, Hu D (2010). Characteristics of prevalence in peripheral arterial disease and correlative risk factors and comorbidities among female natural population in China. VASA.

[4] Selvin E, Erlinger TP (2004). Prevalence of and risk factors for peripheral arterial disease in the United States: results from the National Health and Nutrition examination survey, 1999-2000. CIRCULATION.

[5] Kayaniyil S, Vieth R, Retnakaran R, Knight JA, Qi Y, Gerstein HC, Perkins BA, Harris SB, Zinman B, Hanley AJ (2010). Association of vitamin D with insulin resistance and beta-cell dysfunction in subjects at risk for type 2 diabetes. Diabetes Care.

[6] Pittas AG, Lau J, Hu FB, Dawson-Hughes B (2007). The role of vitamin D and calcium in type 2 diabetes. A systematic review and meta-analysis. J Clin Endocrinol Metab.

[7] Mitri J, Nelson J, Ruthazer R, Garganta C, Nathan DM, Hu FB, Dawson-Hughes B, Pittas AG (2014). Plasma 25-hydroxyvitamin D and risk of metabolic syndrome: an ancillary analysis in the diabetes prevention program. Eur J Clin Nutr.

[8] Faridi KF, Zhao D, Martin SS, Lupton JR, Jones SR, Guallar E, Ballantyne CM, Lutsey PL, Michos ED (2017). Serum vitamin D and change in lipid levels over 5 y: the atherosclerosis risk in communities study. NUTRITION.

[9] Reis JP, Michos ED, von Muhlen D, Miller ER (2008). Differences in vitamin D status as a possible contributor to the racial disparity in peripheral arterial disease. Am J Clin Nutr.

[10] Melamed ML, Muntner P, Michos ED, Uribarri J, Weber C, Sharma J, Raggi P (2008). Serum 25-hydroxyvitamin D levels and the prevalence of peripheral arterial disease: results from NHANES 2001 to 2004. Arterioscler Thromb Vasc Biol.

[11] Zagura M, Serg M, Kampus P, Zilmer M, Eha J, Unt E, Lieberg J, Cockcroft JR, Kals J (2011). Aortic stiffness and vitamin D are independent markers of aortic calcification in patients with peripheral arterial disease and in healthy subjects. Eur J Vasc Endovasc Surg.

[12] Garcia-Canton C, Bosch E, Ramirez A, Gonzalez Y, Auyanet I, Guerra R, Perez MA, Fernandez E, Toledo A, Lago M (2011). Vascular calcification and 25-hydroxyvitamin D levels in non-dialysis patients with chronic kidney disease stages 4 and 5. Nephrol Dial Transplant.

[13] Liew JY, Sasha SR, Ngu PJ, Warren JL, Wark J, Dart AM, Shaw JA (2015). Circulating vitamin D levels are associated with the presence and severity of coronary artery disease but not peripheral arterial disease in patients undergoing coronary angiography. Nutr Metab Cardiovasc Dis.

[14] McDermott MM, Liu K, Ferrucci L, Tian L, Guralnik J, Kopp P, Van Horn L, Liao Y, Green D, Kibbe M (2014). Vitamin D status, functional decline, and mortality in peripheral artery disease. Vasc Med.

[15] Umehara K, Mukai N, Hata J, Hirakawa Y, Ohara T, Yoshida D, Kishimoto H, Kitazono T, Hoka S, Kiyohara Y (2017). Association between serum vitamin D and all-cause and cause-specific death in a general Japanese population- the Hisayama study. Circ J.

[16] De Metrio M, Milazzo V, Rubino M, Cabiati A, Moltrasio M, Marana I, Campodonico J, Cosentino N, Veglia F, Bonomi A (2015). Vitamin D plasma levels and in-hospital and 1-year outcomes in acute coronary syndromes: a prospective study. Medicine (Baltimore).

[17] Gaddipati VC, Bailey BA, Kuriacose R, Copeland RJ, Manning T, Peiris AN (2011). The relationship of vitamin D status to cardiovascular risk factors and amputation risk in veterans with peripheral arterial disease. J Am Med Dir Assoc.

[18] Veronese N, De Rui M, Bolzetta F, Toffanello ED, Coin A, Zambon S, Corti MC, Baggio G, Perissinotto E, Maggi S (2015). Serum 25-Hydroxyvitamin D and the incidence of peripheral artery disease in the elderly: the pro.V.a study. J Atheroscler Thromb.

[19] Rapson IR, Michos ED, Alonso A, Hirsch AT, Matsushita K, Reis JP, Lutsey PL (2017). Serum 25-hydroxyvitamin D is associated with incident peripheral artery disease among white and black adults in the ARIC study cohort. ATHEROSCLEROSIS.

[20] Li DM, Zhang Y, Li Q, Xu XH, Ding B, Ma JH (2016). Low 25-Hydroxyvitamin D level is associated with peripheral arterial disease in type 2 diabetes patients. Arch Med Res.

[21] Zhou W, Ye SD (2015). Relationship between serum 25-hydroxyvitamin D and lower extremity arterial disease in type 2 diabetes mellitus patients and the analysis of the intervention of vitamin D. J Diabetes Res.

[22] Ford ES, Ajani UA, McGuire LC, Liu S (2005). Concentrations of serum vitamin D and the metabolic syndrome among U.S. adults. Diabetes Care.

[23] Forman JP, Giovannucci E, Holmes MD, Bischoff-Ferrari HA, Tworoger SS, Willett WC, Curhan GC (2007). Plasma 25-hydroxyvitamin D levels and risk of incident hypertension. HYPERTENSION.

[24] Martins D, Wolf M, Pan D, Zadshir A, Tareen N, Thadhani R, Felsenfeld A, Levine B, Mehrotra R, Norris K (2007). Prevalence of cardiovascular risk factors and the serum levels of 25-hydroxyvitamin D in the United States: data from the third National Health and Nutrition examination survey. Arch Intern Med.

[25] Equils O, Naiki Y, Shapiro AM, Michelsen K, Lu D, Adams J, Jordan S (2006). 1,25-Dihydroxyvitamin D inhibits lipopolysaccharide-induced immune activation in human endothelial cells. Clin Exp Immunol.

[26] Schleithoff SS, Zittermann A, Tenderich G, Berthold HK, Stehle P, Koerfer R (2006). Vitamin D supplementation improves cytokine profiles in patients with congestive heart failure: a double-blind, randomized, placebo-controlled trial. Am J Clin Nutr.

[27] Wu-Wong JR (2009). Potential for vitamin D receptor agonists in the treatment of cardiovascular disease. Br J Pharmacol.

[28] Li YC, Kong J, Wei M, Chen ZF, Liu SQ, Cao LP (2002). 1,25-Dihydroxyvitamin D (3) is a negative endocrine regulator of the renin-angiotensin system. J Clin Invest.

[29] Zieman SJ, Melenovsky V, Kass DA (2005). Mechanisms, pathophysiology, and therapy of arterial stiffness. Arterioscler Thromb Vasc Biol.

[30] Martini A, Cumarasamy S, Beksac AT, Abaza R, Eun DD, Bhandari A, Hemal AK, Porter JR, Badani KK (2018). A nomogram to predict significant estimated glomerular filtration rate reduction after robotic partial nephrectomy. Eur Urol.

